# Microbiota in the coelomic fluid of two common coastal starfish species and characterization of an abundant *Helicobacter*-related taxon

**DOI:** 10.1038/s41598-017-09355-2

**Published:** 2017-08-18

**Authors:** Satoshi Nakagawa, Hikari Saito, Akihiro Tame, Miho Hirai, Hideyuki Yamaguchi, Takashi Sunata, Masanori Aida, Hisashi Muto, Shigeki Sawayama, Yoshihiro Takaki

**Affiliations:** 1 Laboratory of Marine Environmental Microbiology, Division of Applied Biosciences, Graduate School of Agriculture, Kyoto University, Oiwake-cho, Kitashirakawa, Sakyo-ku, Kyoto 606-8502 Japan; 2 Department of Subsurface Geobiological Analysis and Research (D-SUGAR), Japan Agency for Marine-Earth Science and Technology (JAMSTEC), 2-15 Natsushima-cho, Yokosuka, 273-0061 Japan; 3 Laboratory of Microbiology, Faculty of Fisheries Sciences, Hokkaido University, 3-1-1 Minato-cho, Hakodate, 041-8611 Japan; 4 Department of Technical Services, Marine Works Japan, Ltd., Oppama Higashi-cho, Yokosuka 237-0063 Japan; 5 Research and Development (R&D) Center for Marine Biosciences, Marine Functional Biology Group (MFbio), Japan Agency for Marine-Earth Science and Technology (JAMSTEC), 2-15 Natsushima-cho, Yokosuka, 273-0061 Japan; 6 Leica Microsystems K.K., Osaka Sales Office, Shogyo No. 2 Bldg. 10F, 5-4-9 Toyosaki, Kita-ku, Osaka 531-0072 Japan

## Abstract

Marine invertebrates associate with diverse microorganisms. Microorganisms even inhabit coelomic fluid (CF), namely, the fluid filling the main body cavity of echinoderms. The CF microbiota potentially impacts host health and disease. Here, we analysed the CF microbiota in two common coastal starfish species, *Patiria pectinifera* and *Asterias amurensis*. Although microbial community structures were highly variable among individual starfish, those of *P. pectinifera* were compositionally similar to those in the surrounding seawater. By contrast, many *A. amurensis* individuals harboured unique microbes in the CF, which was dominated by the unclassified *Thiotrichales* or previously unknown *Helicobacter-*related taxon. In some individuals, the *Helicobacter*-related taxon was the most abundant genus-level taxon, accounting for up to 97.3% of reads obtained from the CF microbial community. Fluorescence *in situ* hybridization using a *Helicobacter*-related-taxon-specific probe suggested that probe-reactive cells in *A. amurensis* were spiral-shaped, morphologically similar to known *Helicobacter* species. Electron microscopy revealed that the spiral cells had a prosthecate-like polar appendage that has never been reported in *Helicobacter* species. Although culture of *Helicobacter*-related taxon was unsuccessful, this is the first report of the dominance of a *Helicobacter*-related taxon in invertebrates and non-digestive organs, reshaping our knowledge of the phylogeography of *Helicobacter*-related taxa.

## Introduction

Marine animals live with a diverse array of microorganisms. Like the human microbiome, fish gut bacteria have been intensively studied and suggested to play various significant roles in nutrition, immunity, and defence^1–3^. However, relatively little attention has been paid to the marine invertebrates’ association with bacteria, except those of sponge, corals, squid, and animals endemic to deep-sea vents. In deep-sea vents, episymbiotic or endosymbiotic chemolithoautotrophic bacteria that synthesize organics from CO_2_ provide nutrition to invertebrates, allowing survival in these extreme environments^4^. In coastal areas, gut microbes of invertebrates may provide their host with growth factors, such as vitamins and amino acids, assist in food digestion and produce antimicrobial agents that protect their host from pathogen infections^5^. Notably, microbes have been detected not only from the gut and skin but also from haemolymph, namely, the circulatory fluid that is functionally comparable to the blood and lymph of vertebrates^6, 7^. Playing an analogous role to the endophytes of terrestrial plants, the haemolymph microbiota has important roles in host health and protection^8, 9^. Although understood in many phyla of marine invertebrates, the natural diversity of host-associated microbiota has little been studied in *Echinodermata* members such as starfish, sea urchins, and sea cucumbers^10, 11^.

Coelomic fluid (CF) is the fluid enclosed in the main body cavity of echinoderms, which is equivalent to the haemolymph of invertebrates. The inorganic ion composition of echinoderm CF is slightly different from that of seawater^12^. Echinoderm CF also contains organic compounds, including amino acids, reduced sugars, proteins, lipids and nitrogenous waste^13^. In addition, the CF of echinoderms contains circulating cells, called coelomocytes, whose functions range from metabolite transport to immunity^14^. The coelomocytes phagocytose cell debris and the microorganisms, e.g. microorganisms that invade into the body after injury and autotomy^15^. Despite this immune system activity, the CF of sea cucumber was reported to harbour unique microbial communities including *Sulfurospirillum-* and *Sulfuricurvum-*related taxa within the class *Epsilonproteobacteria* ^16^.

*Asterias* and *Patiria* are common starfish and keystone species in coastal environments in northern Pacific and Atlantic Oceans. Although these starfish are top predators in some marine ecosystems, they are under attack by viruses and bacteria^17^. The body wall and CF of starfish exhibits strong antimicrobial activity, probably due to the saponin glycosides, peptidoglycan recognition proteins, and reactive oxygen species^18–20^. As mentioned above, the starfish CF also contains coelomocytes with phagocytic activity^21, 22^. No commercial benefit of the ability of starfish to synthesize saponins has yet been identified, and starfish are instead increasingly recognized as vermin in fisheries^23, 24^. Although some microorganisms were isolated in pure cultures from starfish body^25^, little is known regarding microbial community structures associated with starfish.

In this study, by the combined use of culture-dependent and -independent methods, we analysed microbial communities associated with common starfish species, *A. amurensis* and *P. pectinifera* (*Echinodermata: Asteroidea*). Specifically, we addressed the questions of whether the CF bacterial composition of *A. amurensis* and *P. pectinifera* differs from that of the surrounding seawater, and whether the composition shows a geographical pattern.

## Results

### Coelomic fluid (CF)

CF samples from common coastal starfish, *A. amurensis* (19 individuals and 1 pooled sample) and *P. pectinifera* (8 individuals and 1 pooled sample), were analysed. The inorganic ion composition of representative CF samples was similar to that of seawater, although pH and most ion concentrations were slightly higher in the CF (Supplementary Table 1).

### Characteristics of sequence data

Microbiota in two common starfish species and their surrounding seawater was studied using 16S rRNA gene amplicon sequencing. A total of 2,473,889 quality-filtered sequence reads were obtained from 34 samples (Supplementary Table 2). Rarefaction curves showed that most starfish samples reached the saturation phase (Supplementary Figure 1). The rarefaction plateaus appeared after fewer reads in *A. amurensis* CF samples, suggesting a lower microbial richness in *A. amurensis* than in *P. pectinifera*. The mean Good’s coverage index of all samples was 0.99, indicating adequate depth of sequencing (Supplementary Table 3).

### Microbiota associated with starfish

**Figure 1:**
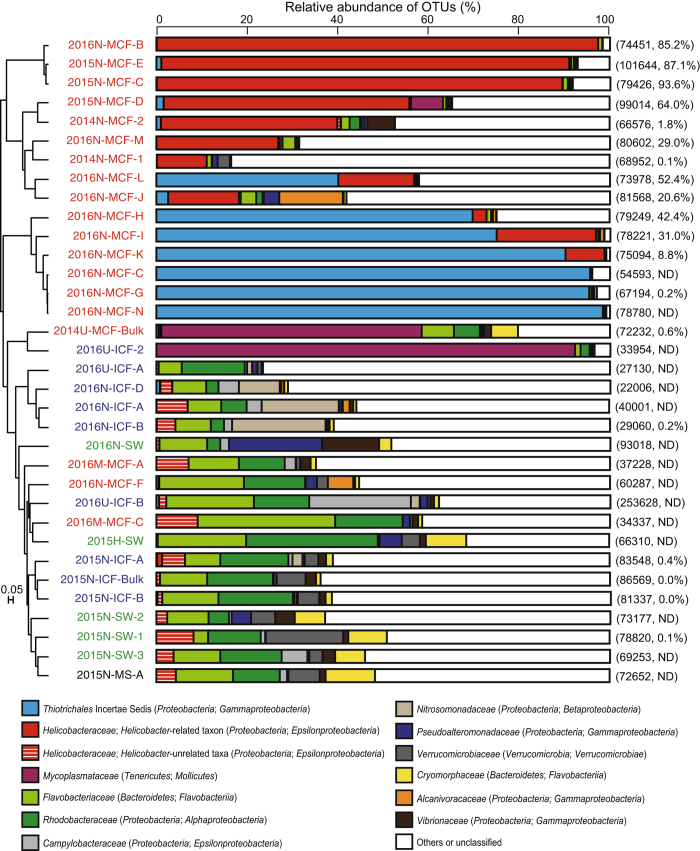
Histogram showing the relative abundance of 16S rRNA gene amplicon reads assigned to family level taxa in the various samples. UPGMA (Unweighted Pair Group Method with Arithmetic mean) clustering based on weighted UniFrac distances is also shown. Each colour on the graph represents a distinct family. Families with >10% relative abundance in any sample are presented, and the rest and unassigned taxa are indicated as ‘others’. The family *Helicobacteraceae* is divided into *Helicobacter-*related taxon and *Helicobacter-*unrelated taxa (mainly *Sulfuricurvum, Sulfurimonas*, and *Sulfurovum* relatives). Sample names represent sampling year, sampling site (N or H, Nemuro City; U or M, Hakodate City), starfish species (*A. amurensis*, M; *P. pectinifera*, I), body parts (CF, coelomic fluid; S, body surface) or seawater (SW), and individual number. “-Bulk” indicates bulk samples. *A. amurensis, P. pectinifera*, seawater, and starfish body surface samples are shown in red, blue, green, and black, respectively. Read numbers and ratios of *Helicobacter*-related taxon against bacteria estimated by quantitative PCR are shown in parentheses on right side of histograms. Detailed taxonomic information is provided in Supplementary Table 5.

**Figure 2:**
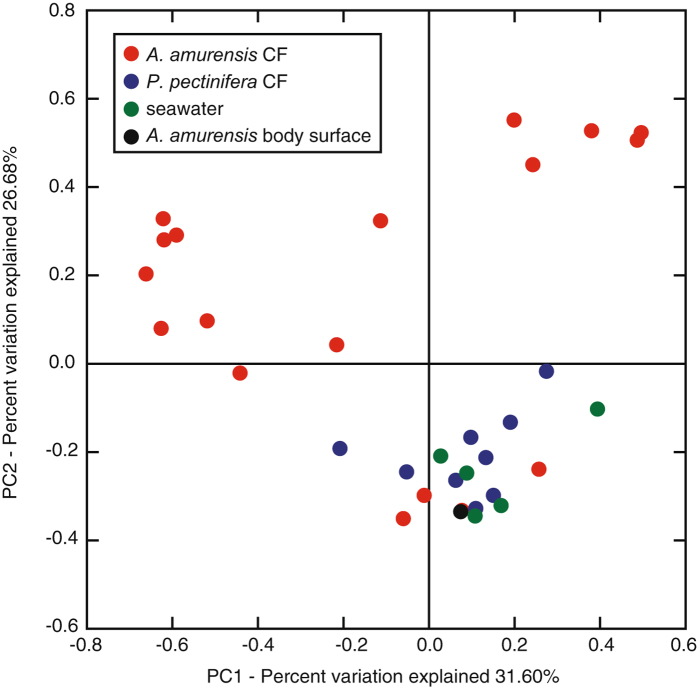
Analysis of microbial communities using principal coordinate analysis (PCoA).

The CF microbiota mostly consisted of bacteria, but markedly varied among individual starfish. Results from the analysis of the alpha diversity metrics on the basis of equal numbers of sequence reads (n = 22,006) confirmed significantly lower microbial richness (Chao1 and observed OTUs) and Shannon’s diversity index for the *A. amurensis* CF compared with both *P. pectinifera* CF and seawater samples (*p* < 0.05 in all cases) (Supplementary Table 3). At the phylum level, the microbial taxonomic composition of CF samples showed a high relative abundance of *Proteobacteria* (59.9% on average), followed by *Bacteroidetes* (8.8%), while some CF samples of *A. amurensis* showed almost complete dominance of *Proteobacteria* (Supplementary Table 4). At the family level, no phylotype was shared across all CF samples with >3.0% relative abundance (Supplementary Table 5), suggesting great variability in the starfish CF microbiota. Nevertheless, the families *Flavobacteriaceae* (*Bacteroidetes*) and *Rhodobacteraceae* (*Alphaproteobacteria*) were frequently recovered from many *P. pectinifera* and several *A. amurensis* individuals. Members of these families were abundantly detected in seawater samples as well (Supplementary Table 5). When all quality-filtered sequence reads from the 5 seawater samples were pooled, *Rhodobacteraceae* was the most abundant, accounting for 12.3%, followed by *Flavobacteriaceae*. The similarity among microbiota within the many *P. pectinifera* CF, some *A. amurensis* CF and seawater was generally supported by cluster analysis (Fig. 1) and principal coordinates analysis (Fig. 2). Members of the family *Mycoplasmataceae* were dominantly recovered from one individual of *P. pectinifera* (sample ID, 2016U-ICF-2) (Fig. 1). At the genus level, the sequences (a total of 4 OTUs from 2016U-ICF-2) were affiliated with the genus *Candidatus* Hepatoplasma. This phylotype was not widely distributed, but was dominantly observed in the CF of *A. amurensis* (2014U-MCF-Bulk) collected from the same sampling site, suggesting the biogeography of starfish CF microbiota. We were generally unable to obtain results for the microbiome on the starfish body surface, probably due to the low microbial biomass there. Nevertheless, the body surface microbiome was successfully studied in one individual of *A. amurensis*, which was similar to that found in seawater (Fig. 1).

As suggested by diversity indices, relatively limited but unique phylotypes were detected in the CF of *A. amurensis*. Although the *A. amurensis* CF microbiota greatly varied between starfish individuals (Fig. 1), the non-parametric multivariate analysis of variance (PERMANOVA) suggested that the microbiota variation resulted from differences in sampling site (at city level) and sampling year (*p* < 0.05). The biogeographical pattern could be due to the small number of *A. amurensis* samples obtained from Hakodate City. The family *Helicobacteraceae* and unclassified *Thiotrichales* were dominantly detected from some *A. amurensis* individuals collected from the coast of Nemuro City in 2015 and 2016 (Fig. 1, Supplementary Table 5). Although these phylotypes were rarely detected from seawater, *P. pectinifera*, or even *A. amurensis* individuals collected from another sampling site, they often showed almost complete dominance in some individuals. Although members of the family *Helicobacteraceae* were detected from both starfish species, their phylogenetic properties were different according to starfish species. All *Helicobacteraceae* abundantly (average >0.5%) found in *P. pectinifera* (3 OTUs) were related to known sulfur-oxidizing bacteria, that is, *Sulfuricurvum, Sulfurimonas* and *Sulfurovum*. Additionally, *Helicobacteraceae* detected in two individuals of *A. amurensis* from Hakodate (4.2% and 6.5% in 2016M-MCF-A and 2016M-MCF-C, respectively) were closely related to *Sulfurovum* species. In contrast, *Helicobacteraceae* in *A. amurensis* from Nemuro were distantly related to known *Helicobacteraceae* members, and their phylogeny could not be inferred in detail from the short-read sequence from MiSeq.

**Figure 3:**
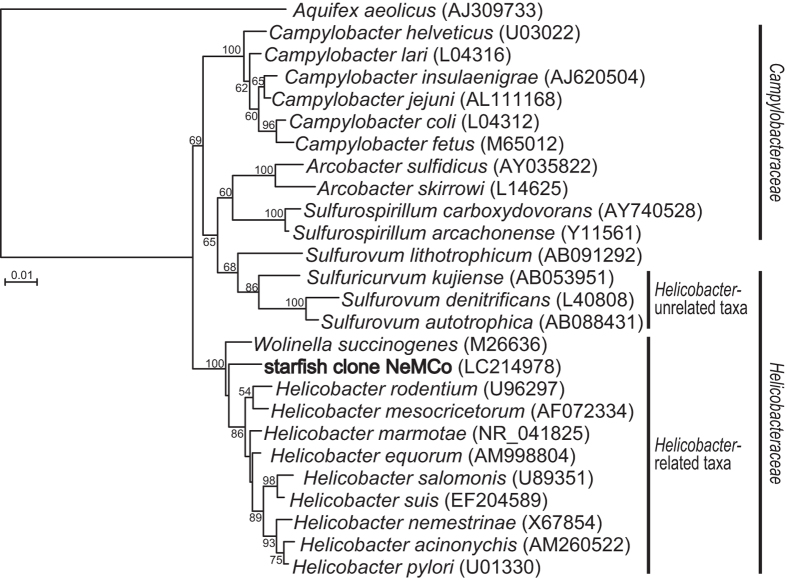
Phylogenetic tree of representative members of the genera *Helicobacter* and *Wolinella* inferred from 16S rRNA gene sequences by the neighbour joining method using 856 homologous sequence positions. The accession numbers are shown in parentheses. Bootstrap values (expressed as percentages of 1,000 replications) higher than 50% are shown at branching points. The sequence found in the CF of *A. amurensis* is shown in bold. The scale bar represents 0.01 substitutions per nucleotide position.

To clarify the phylogenetic position of *Helicobacteraceae* dominantly found in *A. amurensis*, cloning and sequencing of the 16S rRNA gene were performed on one representative sample, 2014N-MCF-2. Half of the cloned sequences (14 out of 28 sequenced clones; one OTU at the 97% cut-off) were affiliated with the family *Helicobacteraceae*, and a BLAST search revealed that the representative clone sequence (1,286 bp in length) was most closely related to *Helicobacter* species, for example, *H. marmotae* and *H. rodentium*, but the identity score was low (89%) (Fig. 3). No known environmental clone sequence was more similar to the *Helicobacteraceae* from *A. amurensis*. The qPCR results generally confirmed the limited but dominant occurrence of the *Helicobacter*-related taxon in *A. amurensis* of Nemuro City (Fig. 1). Likewise, the sequence of almost the full-length (1,348 bp in length) of the 16S rRNA gene of *Thiotrichales* was determined from *A. amurensis* (sample ID, 2016N-MCF-K), which was most closely related to some uncultured clone sequences from seawater (up to 97% identity), but distantly related to validly described species, for example, *Francisella* species (up to 87% identity), sulfur-oxidizing *Piscirickettsiaceae* species (up to 85% identity), and *Candidatus* Endoecteinascidia (up to 84% identity) (Supplementary Figure 2). Even with the almost full-length 16S rRNA gene sequence, the *Thiotrichales* phylotype could not be classified precisely than at the order level.

### Microscopy

**Figure 4:**
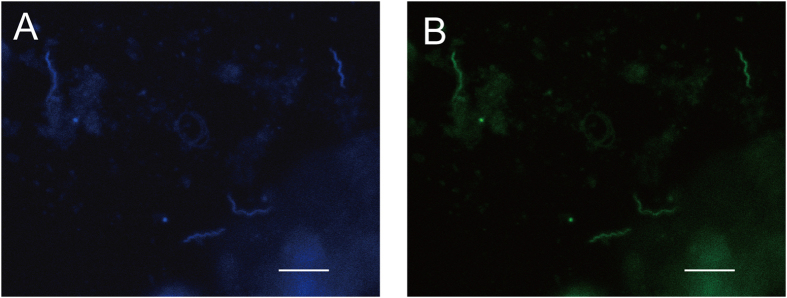
Epifluorescence micrograph of cells binding DAPI (**A**, blue) and the *Helicobacter*-related-taxon-specific probe (**B**, green) in the CF of *A. amurensis* (sample ID, 2015N-MCF-D). Bar, 5 µm.

**Figure 5:**
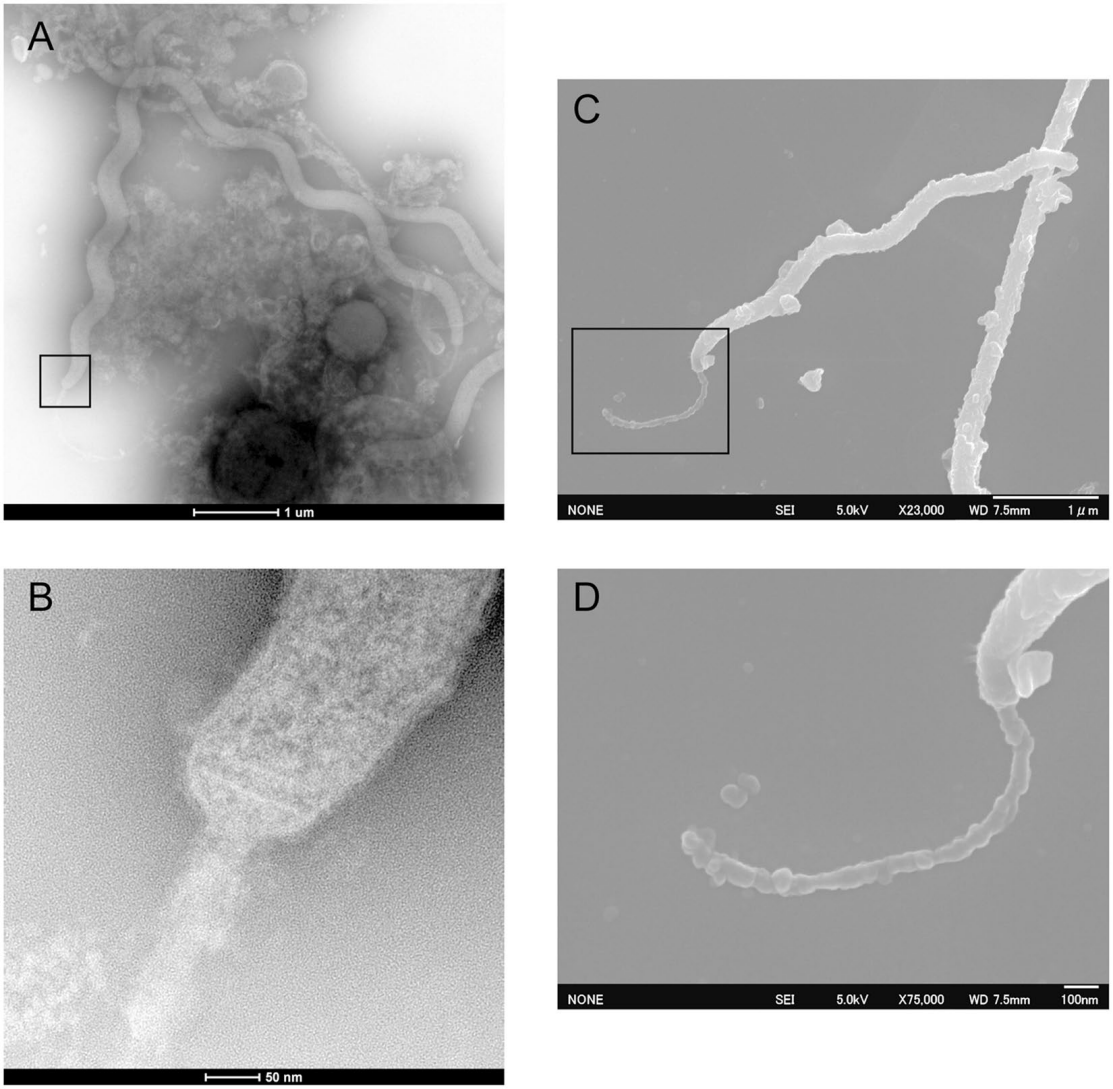
Electron micrographs of the spiral cells found in the CF of *A. amurensis* (sample ID, 2015N-MCF-D). Transmission electron micrographs of negatively stained cells (**A**,**B**). Scanning electron micrographs (**C**,**D**). Magnified views of the polar cell appendage (**B**,**D**).

By FISH analysis with a probe designed for the *Helicobacter*-related taxon, we successfully observed probe-positive cells in the CF of *A. amurensis*. All of the probe-positive cells were spiral-shaped, similar to those of some *Helicobacter* species (Fig. 4). In addition, spiral-shaped cells were observed by transmission electron microscopy (TEM) (Fig. 5A and B) and scanning electron microscopy (SEM) (Fig. 5C and D). Electron microscopy revealed that the spiral-shaped cells had a prosthecate-like polar appendage (Fig. 5B and D), which has never been reported for known *Helicobacter* species.

### Cultivation

We attempted to cultivate the *Helicobacter*-related taxon found in the CF of *A. amurensis* by using a modified version of a medium designed for the selective cultivation of *Helicobacter* species with the addition of basal saline under microaerobic conditions. A total of 34 isolates were obtained from the *A. amurensis* CF (sample ID, 2016N-MCF-J). Phylogenetic analysis based on the 16S rRNA gene sequence suggested that isolates were closely related to the genera *Idiomarina* (17 strains), *Pseudoalteromonas* (11 strains), *Vibrio* (4 strains), *Alcanivorax* (1 strain) and *Thalassospira* (1 strain). The number of colony-forming units (CFUs) was low (≤4.8 × 10^3^ ml^−1^).

### Pathogenicity

To assess the presence of a representative pathogenic gene (*cagA*) and urease gene of *Helicobacter* species in the *Helicobacter*-related taxon that dominated the CF of *A. amurensis*, we performed PCR using specific primers. No amplicon was obtained from all samples (data not shown), suggesting that the risk to humans from the *Helicobacter*-related taxon of starfish is not urgent.

## Discussion

In this study, microbial diversity was assessed for the CF of common coastal starfish species. Given that the antimicrobial activity is well known^15, 26^, the occurrence of microbial diversity within the echinoderm CF might be considered unlikely. We found that the CF of echinoderms could be a reservoir for unique microorganisms including potentially pathogenic and/or symbiotic bacteria. Since the CF microbiota greatly differed among individuals, they are probably not vertically inherited but rather horizontally acquired from the surroundings. This is supported by the general similarity in microbial communities between the CF of *P. pectinifera* and surrounding seawater. Therefore, the bacterial communities in the CF of starfish may be allochthonous, involving the transient passage in and out of surrounding seawater. Many factors potentially influence the composition of starfish CF microbiota, including genetic background, diet, age, stress and environmental factors (e.g. temperature). Nevertheless, *A. amurensis* harboured specific microbiota, namely, *Helicobacter*-related taxon and unclassified *Thiotrichales* members, although this species lives together with *P. pectinifera*. These microorganisms often exhibited almost complete dominance in some individuals. The host specificity of CF microbiota may result from active selection by the host. The dominance of potentially sulfur-oxidizing microbiota might be associated with the starfish feeding behavior; for example, *A. amurensis* has been observed digging up and eating bivalves buried (depth < 10 cm) in sediments^27, 28^. In both starfish species collected in Hakodate City, members of the genus *Candidatus* Hepatoplasma were dominantly detected, although they were not detected in all starfish individuals collected there. Members of this group of bacteria are related to the genus *Mycoplasma*, and have been intensively studied as symbionts living within the hepatopancreas of isopods^29, 30^. Such symbionts are thought to improve the survival of host isopods under low-nutrient conditions^31^. The isopod Hepatoplasma may be inherited from parents to offspring through horizontal transmission^30^. Even in the isopod, however, the infection ratio was reported to significantly vary^30^. Among the sampling sites, the common coastal isopod, *Ligia exotica*, was found to be abundant only in Hakodate City, which might be associated with the near absence of *Candidatus* Hepatoplasma in the starfish CF from Nemuro City.

Mainly from the *A. amurensis* CF collected from Nemuro City in 2016, unclassified members of the order *Thiotrichales* were dominantly detected. Although it was not possible to classify them precisely, the 16S rRNA gene sequence was distantly related to those of *Francisella*, sulfur-oxidizing *Piscirickettsiaceae* and *Candidatus* Endoecteinascidia. Members of the genus *Francisella* are strictly aerobic, facultatively intracellular heterotrophs, which include the fatal human pathogen, *F. tularensis* ^32^. *Francisella* members also infect various fish and shellfish species, and are known as the causative agent of severe disease^33^. In Japan, *F. halioticida* caused the mass mortality of the abalone *Haliotis gigantea* ^33, 34^. In contrast, in sulfidic marine environments, symbiotic sulfur-oxidizing *Thiotrichales* both participate in the detoxification of sulfide and heavy metals and fix carbon for their host invertebrates^4^. *Candidatus* Endoecteinascidia is a commercially important symbiotic bacterium of the Caribbean mangrove tunicate *Ecteinascidia turbinata* ^35^. This symbiont produces an anti-tumour compound, ET-743, and potentially protects the host from predators^36^. Precise characterization of the physiology of the starfish *Thiotrichales* appears to be necessary, considering its potential positive and negative impacts on fisheries^23, 24^.

Among members of the class *Epsilonproteobacteria, Arcobacter* species (family *Campylobacteraceae*) were previously detected in the gut of sea urchins^37, 38^. In this study, some *P. pectinifera* individuals harboured close relatives of known species of the genus *Arcobacter* (up to 22.4% in relative abundance of sequences). In addition, the *Sulfuricurvum*-related OTU detected in *P. pectinifera* (up to 6.7%) was closely related to the phylotype previously detected in the CF of sea cucumber^16^, suggesting a potential similarity in settings among sea urchin guts, sea cucumber CF and *P. pectinifera* CF. Although these *Epsilonproteobacteria* were scarcely detected in *A. amurensis* CF, previously unknown *Helicobacter*-related taxon was mainly detected from *A. amurensis* collected from Nemuro City in 2015.

The 16S rRNA gene sequence of *Helicobacter*-related taxon was most closely related to *Helicobacter rodentium* and *H. marmotae*, which were respectively isolated from laboratory mice^39^ and livers of woodchucks and intestines of cats^40^. Members of the genus *Helicobacter* are diverse in vertebrate hosts, infecting the digestive tracts of a wide array of mammals and birds^41^. Recently, *Helicobacter* species were also found in reptiles^42^. Well-characterized *Helicobacter*-related taxa include *Wolinella* species, which are the cattle rumen-associated non-pathogenic members of the *Epsilonproteobacteria* ^43^. The *HelicobacterWolinella* group has never been isolated from invertebrates^44^. Although some studies detected *Helicobacter* species in coastal seawater^45, 46^, these microbes were probably in a dormant state. Although the starfish *Helicobacter*-related taxon was not successfully cultured in this study, this is the first report to describe the dominance of *Helicobacter*-related taxon in invertebrates and non-digestive organs. The failure of cultivation of the *Helicobacter*-related taxon from *A. amurensis* might suggest that it had lost the ability to grow outside the host or differs physiologically from known *Helicobacter* species. Considering that many marine *Helicobacteraceae* species have the ability to oxidize sulfide^4^, the *Helicobacter*-related taxon might aid sulfide detoxification, as hypothesized for unclassified *Thiotrichales*. Although virulence gene *cagA* and the urease gene were not detected by PCR, primers used in this study were designed for *H. pylori*. In addition, some *Helicobacter* species, including *H. pylori* strains, are known to lack these genes^47^. More genomic and physiological characterization of the *Helicobacter-*related taxon found in *A. amurensis* are necessary in the future. Nevertheless, the successful isolation of diverse bacteria suggested the suitability of the main body cavity of starfish for habitation by microbes. Previous comparative genomic analysis revealed that epsilonproteobacterial autotrophy in deep-sea vents has provided the core of virulence for important human/animal pathogens including *Helicobacter* ^48, 49^. The occurrence of *Helicobacter*-related taxon within the coastal echinoderm CF may fill the evolutionary gap between the deep-sea vents and terrestrial vertebrate intestinal habitats. In this context, *Echinodermata* are amongst the most abundant animals in the deep-sea, and our findings warrant the assessment of deep-sea echinoderm microbiota.

The CF microbiota potentially have adverse effects on the health of starfish. In contrast, the bacteria may confer some competitive advantages to starfish by allowing starfish survival under nutrient stress, producing bioactive compounds to repel predators, and/or detoxifying sulfide. Considering their resistance to cultivation, further studies using metagenomics or single-cell genomics are required to elucidate the precise nature of the starfish-bacteria association. Such studies should be combined with a functional approach in which controlled *in vivo* experiments are used to determine the degree of dependence of the starfish on its microbiota, e.g. during regeneration.

## Methods

### Sampling

Starfish individuals were collected from piers using a net in coastal areas of two different cities in Hokkaido, Japan: Nemuro City (43.2–3′N, 145.58–59′E) and Hakodate City (41.8–9′N, 140.6–9′E). Two sites in each city were studied. At the sampling sites, *A. amurensis* and *P. pectinifera* live together, although *A. amurensis* was rarely found in the area of Nemuro with turbid seawater. All starfish individuals appeared healthy, and no evidence of autotomy was observed. For some individuals, outer surfaces of the body wall were sampled before sampling the CF, using sterile cotton-tipped swabs (Osakimedical, Nagoya, Japan). A single arm tip was cut using sterilized scissors to pour CF into a sterile tube. The volume of CF fluctuated markedly among the individuals, but at least 5 ml was used for the analysis described below. Although over 100 ml of CF was collected from an individual in some *A. amurensis*, the CF from small individuals was pooled and then analyzed (sample IDs: 2014U-MCF-Bulk and 2015N-ICF-Bulk). For FISH and electron microscopy, the CF microbiota was fixed in 4% paraformaldehyde immediately after the CF collection. Digestive glands were also collected after dissection with sterilized scissors. Seawater samples (100-500 ml) were collected using a VanDorn sampler and immediately filtered through 0.22-µm-pore-size polyethersulfone filters. All samples (CF, filters, and digestive glands) were kept on ice and transported to the laboratory as quickly as possible. Because no laboratory facility was available in Nemuro, the most eastern city of Hokkaido, sample transportation took up to 30 hours after sampling (up to 10 hours for samples from Hakodate). Details of samples are summarized in Supplementary Table 2.

Immediately upon arrival at the laboratory, host cells in the CF were pelleted at 800 g for 5 min^50^, and the supernatant containing microbial cells was further centrifuged at 21,500 g for 10 min. The obtained supernatants were then subjected to ion chromatography. The microbial cell pellets and filters from seawater samples were kept at −80 °C for use in DNA extraction. For FISH and electron microscopy, fixed cells were washed three times with PBS, and preserved in 50% (v/v) ethanol in PBS at −30 °C.

### Ion chromatography

Anion and cation concentrations were determined by ion chromatography using a Shim-pack IC-A3 column (Shimadzu, Kyoto, Japan) and a Shim-pack IC-C3 column (Shimadzu), respectively. The Shimadzu HPLC CBM-20A system controller and CDD-10AVP conductivity detector were used.

### DNA analysis

Genomic DNA was extracted from cell pellets of CF samples, seawater filters, and swabs using the Ultraclean Microbial DNA isolation kit (MoBio Laboratories, Carlsbad, CA) following the manufacturer’s instructions. For digestive gland samples, DNeasy blood and tissue kit (Qiagen, Valencia, CA) was used, but no microbial components were detected (data not shown), potentially due to the presence of strong DNase and/or PCR inhibitors. The V4 regions of 16S rRNA genes were amplified using the LA Taq (TaKaRa Bio Inc., Otsu, Japan). Negative controls without template DNA were included in each PCR trial. The first PCR step (20-35 cycles) was performed using primers 515F (5′-ACACTCTTTCCCTACACGACGCTCTTCCGATCTGTGCCAGCMGCCGCGGTAA-3′) and 806R (5′-GTGACTGGAGTTCAGACGTGTGCTCTTCCGATCTGGACTACHVGGGTWTCTAAT-3′). These primers are suitable for the amplification of both bacterial and archaeal DNA. The amplified products from the minimum PCR cycles with visible PCR product were used for a second round of PCR after the purification with AMPure XP (Beckman Coulter, Mississauga, Canada). The second amplification step (10 cycles) used the first PCR product as a template and was performed using primers with a tag sequence. After the second PCR, amplicons were purified again using AMPure XP. After measuring the concentrations of each PCR product using the Qubit fluorometer (Thermo Fisher Scientific Inc., MA, USA), amplicons were sent to FASMAC (Atsugi, Japan) for Illumina sequencing (MiSeq). The raw sequencing data have been submitted at GenBank/EMBL/DDBJ under accession number DRA005566.

Pair-end reads were merged with PEAR^51^. Mitochondrial sequences were removed from the data set by mapping the reads against mitochondrial genomes of *A. amurensis* and *P. pectinifera* using Bowtie 2^52^. After the removal of primers, low-quality (Q score <30 in more than 3% of sequences) and short (<150 bp) reads were removed using a custom perl script. Sequences (253 bp average) were processed using the QIIME software package^53^. After the removal of chimeric sequences using Usearch61^54^ in QIIME, OTUs were selected at the 97% similarity level using UCLUST^54^ and were subsequently assigned to a taxon (at phylum, class, order, family, and genus levels) by comparison with the non-redundant 16S rRNA small subunit SILVA128 database^55^, using the RDP classifier^56^ with the default parameters. Alpha and beta diversity analyses were performed on the rarefied OTU table at 22,006 sequences per sample in QIIME. Alpha diversity indices were compared between starfish species with a non-parametric t-test (Monte Carlo, 999 permutations) in QIIME. An unweighted pair group method algorithm (UPGMA) dendrogram was made in QIIME using a weighted UniFrac distance matrix^57^. Similarly, principal coordinate analysis (PCoA) was performed in QIIME. Differences in CF microbial community by sampling site and year were evaluated using permutational multivariate analysis of variance (PERMANOVA) based on weighted UniFrac distance matrix in QIIME.

To obtain nearly the full-length sequence of the 16S rRNA gene, cloning and sequencing were performed using the 27F and 1492R primers, as previously described^58^ for two samples. Phylogenetic analysis was performed using ARB software^59^ as described previously^58^. Furthermore, real-time PCR was performed on all DNA samples by using the *Helicobacter*-related-taxon-specific primer Hel398F (5′-GAGGATGACGGCTTTCGAGT-3′) and the universal primer 533R (5′-TTACCGCGGCKGCTGRCAC-3′)^60^. Targets of these primers were checked using the Probe Match tool of ARB software^59^. The Hel398F primer had at least three mismatches to non-target sequences. The SYBR Premix Ex Taq kit (TaKaRa) was used with the Thermal Cycler Dice Real Time System Lite (TaKaRa). The qPCR mixtures contained 12.5 µl of SYBR Premix Ex Taq II, 2 µl of each forward and reverse primer (10 pmol ul^−1^), 6.5 µl of PCR grade water, and 2 µl of the DNA template. Purified PCR products amplified from a CF DNA sample (2015N-MCF-E) with 338F^61^/533R primers and 398F/533R primers were used as quantification standards of bacteria and *Helicobacter*-related taxon, respectively. Concentrations of the standards were measured fluorometrically with Qubit (Thermo Fisher Scientific). The PCR conditions were 95 °C for 1 min, followed by 40 cycles of 95 °C for 5 s and 60 °C for 30 s. Dissociation curve analysis and gel electrophoresis were performed for quality assurance. The 16S rRNA gene sequences of *Helicobacter*-related taxon and unclassified *Thiotrichales* reported in this study are available under accession numbers LC214978 and LC278460, respectively.

To assess the presence of pathogenic genes in the starfish *Helicobacter*-related taxon, PCR was performed using primer sets designed for *cagA* ^62^ and *ureA* ^63^. Genomic DNA of *H. pylori* strain NCTC 11637 was purchased from American Type Culture Collection (Manassas, VA) and used as a positive control.

### Cultivation

To cultivate microorganisms found in the CF of *A. amurensis*, some CF samples were transferred to the solid medium, modified Columbia horse blood agar HP (CHBHP). The medium (per litre) contains yeast extract 2 g, peptone 0.5 or 0.1 g, agar 12 g, horse blood 100 ml, and the *Helicobacter pylori* selective supplement 2 vials (Thermo Fisher Scientific). The medium composition was slightly modified from CHBHP medium^64^. Two-thirds diluted MMJ synthetic seawater^65^ or the supernatant of CF (after 21,500 g for 10 min) was used as the basal saline. The anaeropack (Mitsubishi Gas Co., Ltd.) was used for microaerobic conditions. Plates were incubated at the *in-situ* temperature (17 °C).

### Microscopy

Fluorescence *in-situ* hybridization (FISH) was performed on one representative CF sample, 2015N-MCF-D (fixed at the sampling site), as described previously^66^, using *Helicobacter-*specific probe Hel395F with Alexa Fluor 488 (5′-CKAAAWCCKTCATCCTCCAC-3′) (designed in this study). Formamide concentration (set to 30%) and specificity was predicted using mathFISH^67^. After 4′-6-diamidino-2-phenylindole (DAPI) staining, samples were observed under a confocal laser scanning microscope (TCS-SP8; Leica Microsystems, Wetzlar, Germany) equipped with filters for Alexa Fluor 488 (excitation 488 nm; emission 500-550 nm) and DAPI (excitation 405 nm; emission 423-456 nm). Non338 was used as a negative control probe^68^. For transmission electron microscopy, the cell pellet from a CF sample, 2015N-MCF-D (fixed at the sampling site), was stained with 1% (v/v) phosphotungstic acid containing 0.01% (w/v) sodium azide and 0.4% (w/v) sucrose (pH7.6). Micrographs were captured using a Technai G^2^ 20 electron microscope (FEI, Hillsboro, OR) operated at 200 kV. Cells were also observed with a JEOL JSM-6700F scanning electron microscope.

## Electronic supplementary material

Supplementary tables and figure
