# Supplementary material for: Microbiota in the coelomic fluid of two common coastal starfish species and characterization of an abundant Helicobacter-related taxon
Source: Sci Rep. 2017 Aug 18;7:8764. doi: 10.1038/s41598-017-09355-2 (PMC5562702; doi:10.1038/s41598-017-09355-2)
Supplement: Supplementary file 1 — Supplementary tables and figure [file 41598_2017_9355_MOESM1_ESM.pdf]

Microbiota in the coelomic fluid of two common coastal starfish species and characterization of an abundant *Helicobacter*-related taxon

Satoshi Nakagawa[1,2,3]*, Hikari Saito[1], Akihiro Tame[4], Miho Hirai[5], Hideyuki Yamaguchi[6], Takashi Sunata[3], Masanori Aida[3], Hisashi Muto[1], Shigeki Sawayama[1], Yoshihiro Takaki[2]

[1]Laboratory of Marine Environmental Microbiology, Division of Applied Biosciences, Graduate School of Agriculture, Kyoto University, Oiwake-cho, Kitashirakawa, Sakyo-ku, Kyoto 606-8502, Japan
[2]Department of Subsurface Geobiological Analysis and Research (D-SUGAR), Japan Agency for Marine-Earth Science and Technology (JAMSTEC), 2-15 Natsushima-cho, Yokosuka 273-0061, Japan
[3]Laboratory of Microbiology, Faculty of Fisheries Sciences, Hokkaido University, 3-1-1 Minato-cho, Hakodate 041-8611, Japan
[4]Department of Technical Services, Marine Works Japan, Ltd., Oppama Higashi-cho, Yokosuka 237-0063, Japan
[5]Research and Development (R&D) Center for Marine Biosciences, Marine Functional Biology Group (MFbio), Japan Agency for Marine-Earth Science and Technology (JAMSTEC), 2-15 Natsushima-cho, Yokosuka 273-0061, Japan
[6]Leica Microsystems K.K., Osaka Sales Office, Shogyo No. 2 Bldg. 10F, 5-4-9 Toyosaki, Kita-ku, Osaka 531-0072, Japan

Running title: Microbes within starfish coelomic fluid
Keywords: Starfish / Coelomic fluid / *Helicobacter*

Submitted to *Scientific Reports*

*Corresponding author.
Phone: +81-75-753-6355; E-mail: nsatoshi@kais.kyoto-u.ac.jp

Supplementary Table 1. Ion composition and pH of samples used in this study.

	Cl^- (mM)	Br^- (mM)	SO_4^{2-} (mM)	Na^+ (mM)	K^+ (mM)	Mg^{2+} (mM)	Ca^{2+} (mM)	pH
CF of *A. amurensis*								
2015N-MCF-C	554.6	1.0	23.2	567.9	13.7	55.3	15.6	7.9
2015N-MCF-D	541.1	1.0	23.7	564.4	13.2	50.5	14.1	7.7
2015N-MCF-E	553.1	1.0	20.3	527.6	12.4	49.9	15.9	7.7
CF of *P. pectinifera*								
2016U-ICF-A	538.2	1.0	21.5	544.1	17.3	46.3	13.4	7.7
2015N-ICF-B[a]	541.7	0.9	21.5	549.4	15.1	52.1	16.7	7.7
2015N-ICF-Bulk[a]	543.4	1.0	21.6	576.1	12.4	54.9	16.0	8.1
Seawater								
2015N-SW-3[a]	446.4	0.8	22.4	496.7	10.3	41.6	11.8	7.7
2015H-SW	559.3	0.9	23.5	550.1	13.3	52.2	14.0	7.1
2015N-SW-1	566.5	0.9	23.2	482.2	13	49.9	12.0	6.4
2015N-SW-2	505.5	0.8	21.7	492.0	12.3	48.9	13.0	6.4

[a]Sampled at the turbid seawater area.

Supplementary Table 2. Details of samples used in this study.

Sample ID	Sample description	Volume (ml)	Sampling date (yyyy-mm-dd)	DNA conc (ng µl^{-1})[a]	A260 /280[b]	Sampling location[c]
2015N-MCF-E	coelomic fluid of *A. amurensis*	110	2015-09-08	0.8	1.8	Japan: Hokkaido, Nemuro, Nemuro
2015N-MCF-D	coelomic fluid of *A. amurensis*	145	2015-09-08	0.7	1.8	Japan: Hokkaido, Nemuro, Nemuro
2016N-MCF-M	coelomic fluid of *A. amurensis*	12	2016-09-01	2.0	ND	Japan: Hokkaido, Nemuro, Nemuro
2016N-MCF-B	coelomic fluid of *A. amurensis*	23	2016-08-31	0.3	1.8	Japan: Hokkaido, Nemuro, Nemuro
2015N-MCF-C	coelomic fluid of *A. amurensis*	15	2015-09-07	ND	1.8	Japan: Hokkaido, Nemuro, Nemuro
2014N-MCF-1	coelomic fluid of *A. amurensis*	43	2014-09-09	1.0	1.8	Japan: Hokkaido, Nemuro, Nemuro
2014U-MCF-Bulk	coelomic fluid of *A. amurensis* (pooled from 2 individuals)	40	2014-10-01	<0.2	1.9	Japan: Hokkaido, Hakodate, Usujiri
2016N-MCF-F	coelomic fluid of *A. amurensis*	6	2016-09-01	0.7	1.9	Japan: Hokkaido, Nemuro, Nemuro
2014N-MCF-2	coelomic fluid of *A. amurensis*	45	2014-09-09	0.9	2.0	Japan: Hokkaido, Nemuro, Nemuro
2016M-MCF-A	coelomic fluid of *A. amurensis*	42	2016-05-28	4.6	2.0	Japan: Hokkaido, Hakodate, Moheji
2016M-MCF-C	coelomic fluid of *A. amurensis*	25	2016-05-29	<0.2	1.6	Japan: Hokkaido, Hakodate, Moheji
2016N-MCF-L[d]	coelomic fluid of *A. amurensis*	17	2016-09-01	0.6	2.1	Japan: Hokkaido, Nemuro, Nemuro
2016N-MCF-C	coelomic fluid of *A. amurensis*	23	2016-08-31	3.4	1.9	Japan: Hokkaido, Nemuro, Nemuro
2016N-MCF-G	coelomic fluid of *A. amurensis*	17	2016-09-01	4.9	2.0	Japan: Hokkaido, Nemuro, Nemuro
2016N-MCF-I	coelomic fluid of *A. amurensis*	12	2016-09-01	0.8	2.0	Japan: Hokkaido, Nemuro, Nemuro
2016N-MCF-K	coelomic fluid of *A. amurensis*	17	2016-09-01	14.5	1.9	Japan: Hokkaido, Nemuro, Nemuro
2016N-MCF-J	coelomic fluid of *A. amurensis*	16	2016-09-01	0.6	2.2	Japan: Hokkaido, Nemuro, Nemuro
2016N-MCF-N	coelomic fluid of *A. amurensis*	5	2016-09-01	2.0	1.9	Japan: Hokkaido, Nemuro, Nemuro
2016N-MCF-H	coelomic fluid of *A. amurensis*	5	2016-09-01	1.7	2.0	Japan: Hokkaido, Nemuro, Nemuro
2015N-ICF-Bulk[d]	coelomic fluid of *P. pectinifera* (pooled from <10 individuals)	50	2015-09-08	0.9	1.9	Japan: Hokkaido, Nemuro, Nemuro
2016N-ICF-A	coelomic fluid of *P. pectinifera*	18	2016-09-01	0.1	1.7	Japan: Hokkaido, Nemuro, Nemuro
2015N-ICF-B[d]	coelomic fluid of *P. pectinifera*	17	2015-09-08	1.0	1.4	Japan: Hokkaido, Nemuro, Nemuro
2015N-ICF-A[d]	coelomic fluid of *P. pectinifera*	18	2015-09-08	1.5	1.3	Japan: Hokkaido, Nemuro, Nemuro
2016N-ICF-B	coelomic fluid of *P. pectinifera*	30	2016-09-01	0.8	1.7	Japan: Hokkaido, Nemuro, Nemuro
2016U-ICF-B	coelomic fluid of *P. pectinifera*	23	2016-05-27	3.2	1.9	Japan: Hokkaido, Hakodate, Usujiri
2016U-ICF-A	coelomic fluid of *P. pectinifera*	18	2016-05-27	<0.2	1.9	Japan: Hokkaido, Hakodate, Usujiri
2016N-ICF-D	coelomic fluid of *P. pectinifera*	20	2016-09-01	4.0	1.9	Japan: Hokkaido, Nemuro, Nemuro
2016U-ICF-2	coelomic fluid of *P. pectinifera*	18	2016-05-27	1.2	1.7	Japan: Hokkaido, Hakodate, Usujiri
2016N-SW	seawater	500	2016-09-01	<0.2	1.3	Japan: Hokkaido, Nemuro, Nemuro
2015N-SW-2	seawater	200	2015-09-08	<0.2	1.7	Japan: Hokkaido, Nemuro, Nemuro
2015N-SW-3[d]	seawater	100	2015-09-08	<0.2	1.7	Japan: Hokkaido, Nemuro, Nemuro
2015N-SW-1	seawater	200	2015-09-08	0.2	1.5	Japan: Hokkaido, Nemuro, Nemuro
2015H-SW	seawater	200	2015-09-08	1.1	1.9	Japan: Hokkaido, Nemuro, Hanasaki
2015N-MS-A	body surface of *Asterias amurensis*	-	2015-09-07	<0.2	1.5	Japan: Hokkaido, Nemuro, Nemuro

ND, not determined.

[a]Measured with a Qubit fluorometer (Thermo Fisher Scientific). All DNA solutions were 50 µl. The DNA from CF must include both microbial DNA and starfish DNA.

[b]Measured with a Nanodrop (Thermo Fisher Scientific).

[c]The direct distance between Nemuro and Hanasaki is 6 km. Moheji and Usujiri is approximately 35 km.

[d]Collected from the turbid seawater area.

Supplementary Table 3. Alpha diversity (mean ± SEM) of microbial communities based on rarefied OTU table (22,006 sequences per sample) and Good's coverage.

	Shannon index	Chao1	Observed OTUs	Good's coverage
Coelomic fluid (*A. amurensis*)				
2014N-MCF-1	3.48 ± 0.00	569 ± 31	203 ± 3	1.00
2014N-MCF-2	3.66 ± 0.00	1832 ± 39	863 ± 5	0.99
2014U-MCF-Bulk	4.28 ± 0.01	1722 ± 36	970 ± 5	0.99
2015N-MCF-C	1.29 ± 0.01	1357 ± 32	721 ± 7	0.99
2015N-MCF-D	2.49 ± 0.01	1678 ± 30	881 ± 6	0.99
2015N-MCF-E	1.31 ± 0.01	1520 ± 26	797 ± 8	0.99
2016M-MCF-A	5.85 ± 0.01	1606 ± 58	574 ± 5	0.99
2016M-MCF-C	6.31 ± 0.00	965 ± 32	400 ± 3	0.98
2016N-MCF-F	7.68 ± 0.00	3204 ± 49	1253 ± 6	0.98
2016N-MCF-G	0.53 ± 0.00	328 ± 10	189 ± 2	1.00
2016N-MCF-H	1.51 ± 0.00	239 ± 11	167 ± 3	1.00
2016N-MCF-I	1.13 ± 0.00	260 ± 13	150 ± 2	1.00
2016N-MCF-J	3.45 ± 0.01	664 ± 15	344 ± 3	1.00
2016N-MCF-K	0.59 ± 0.00	160 ± 9	92 ± 2	1.00
2016N-MCF-M	1.90 ± 0.00	664 ± 17	386 ± 2	1.00
2016N-MCF-N	0.21 ± 0.00	242 ± 17	111 ± 3	1.00
2016N-MCF-L	1.95 ± 0.00	281 ± 17	144 ± 2	1.00
2016N-MCF-B	0.34 ± 0.00	163 ± 4	116 ± 1	1.00
2016N-MCF-C	0.48 ± 0.00	158 ± 8	96 ± 1	1.00
Coelomic fluid (*P. pectinifera*)				
2015N-ICF-A	8.40 ± 0.00	5448 ± 67	2881 ± 10	0.97
2015N-ICF-B	8.93 ± 0.00	4480 ± 63	2469 ± 6	0.97
2015N-ICF-Bulk	8.40 ± 0.00	3467 ± 56	1659 ± 5	0.98
2016N-ICF-A	5.18 ± 0.01	1845 ± 62	772 ± 4	0.99
2016N-ICF-B	4.74 ± 0.00	1515 ± 29	890 ± 2	0.99
2016N-ICF-D	4.12 ± 0.00	1018.5 ± 0	466 ± 0	0.99
2016U-ICF-A	3.85 ± 0.00	2378 ± 65	631 ± 2	0.98
2016U-ICF-B	6.60 ± 0.00	1931 ± 33	864 ± 4	0.99
2016U-ICF-2	0.96 ± 0.00	953 ± 22	441 ± 2	0.99
Starfish body surface (*A. amurensis*)				
2015N-MS-A	7.83 ± 0.00	3789 ± 36	2021 ± 8	0.99
Seawater				
2015N-SW-1	7.43 ± 0.01	3853 ± 42	2017 ± 10	0.98
2015N-SW-2	8.01 ± 0.01	2699 ± 42	1783 ± 6	0.99
2015N-SW-3	8.02 ± 0.01	2640 ± 25	1724 ± 4	0.98
2015H-SW	6.80 ± 0.01	3952 ± 84	1524 ± 8	0.97
2016N-SW	5.18 ± 0.00	625 ± 21	252 ± 3	1.00

Supplementary Table 4. Abundance (%) of major phyla in each samples. Phyla with >1.0% relative abundance in at least one sample are shown. Minor or unassigned phyla are shown as "Unassigned and other phyla".

	CF (*A. amurensis*)																		
	2014N-MCF-1	2015N-MCF-E	2015N-MCF-D	2016N-MCF-M	2016N-MCF-B	2015N-MCF-C	2014U-MCF-Bulk	2016N-MCF-F	2014N-MCF-2	2016M-MCF-A	2016M-MCF-C	2016N-MCF-C	2016N-MCF-I	2016N-MCF-N	2016N-MCF-H	2016N-MCF-K	2016N-MCF-J	2016N-MCF-L	2016N-MCF-G
Bacteria; *Proteobacteria*	24.2	94.6	60.0	30.1	98.6	92.4	22.1	62.2	54.6	44.7	53.3	97.3	99.0	99.2	75.8	99.3	49.1	59.3	98.5
Bacteria; *Tenericutes*	0.0	0.1	7.1	0.6	0.0	0.1	57.5	0.0	0.2	0.0	0.2	0.0	0.0	0.0	0.0	0.0	0.4	0.0	0.0
Bacteria; *Bacteroidetes*	1.2	1.2	1.4	3.4	1.2	1.5	14.6	26.2	2.8	17.1	36.5	0.2	0.7	0.5	1.4	0.4	5.7	0.4	0.7
Bacteria; *Verrucomicrobia*	6.3	0.5	0.3	0.2	0.0	0.3	0.7	3.2	1.0	2.2	1.3	0.0	0.0	0.1	0.1	0.0	0.0	0.0	0.1
Bacteria; *Cyanobacteria*	0.0	0.0	0.1	0.0	0.0	0.1	0.5	2.3	0.4	0.6	1.7	0.0	0.0	0.0	0.0	0.0	0.2	0.0	0.0
Bacteria; *Planctomycetes*	3.0	0.4	0.4	0.0	0.0	0.4	0.5	1.2	0.5	0.6	0.0	0.1	0.0	0.0	0.0	0.0	0.2	0.0	0.0
Bacteria; *Actinobacteria*	4.7	0.8	0.7	0.1	0.0	1.0	0.3	1.9	0.5	0.3	0.8	0.7	0.0	0.0	0.1	0.0	0.1	0.0	0.0
Bacteria; *Firmicutes*	3.3	0.1	0.1	0.2	0.0	0.1	0.5	0.4	3.9	0.9	0.5	0.3	0.0	0.0	0.0	0.0	0.1	0.2	0.1
Bacteria; *Deinococcus-Thermus*	0.0	0.0	0.0	0.0	0.0	0.0	0.2	0.5	0.4	0.0	0.7	0.0	0.0	0.0	0.0	0.0	0.1	0.1	0.0
Archaea; *Euryarchaeota*	0.0	0.0	0.0	0.0	0.0	0.0	0.0	0.0	0.0	0.0	0.0	0.0	0.0	0.0	0.0	0.0	0.0	0.0	0.0
Unassigned and other phyla	57.1	0.7	28.3	64.6	0.1	2.7	2.2	1.3	34.9	32.6	3.5	1.3	0.2	0.1	22.6	0.2	44.0	40.0	0.5

	CF (*P. pectinifera*)									Seawater					Starfish body surface
	2015N-ICF-Bulk	2016N-ICF-A	2015N-ICF-B	2015N-ICF-A	2016N-ICF-B	2016U-ICF-B	2016U-ICF-A	2016N-ICF-D	2016U-ICF-2	2016N-SW	2015N-SW-3	2015N-SW-1	2015H-SW	2015N-SW-2	2015N-MS-A
Bacteria; *Proteobacteria*	40.6	51.7	44.9	51.6	41.9	63.3	30.5	32.9	5.2	64.2	60.3	51.0	52.5	49.8	47.8
Bacteria; *Tenericutes*	0.0	0.0	0.0	0.0	0.0	0.1	0.2	0.0	92.3	0.0	0.0	0.1	0.0	0.1	0.1
Bacteria; *Bacteroidetes*	21.2	11.1	23.3	17.6	12.0	25.0	6.0	10.5	1.5	22.2	25.2	21.3	36.9	30.0	34.9
Bacteria; *Verrucomicrobia*	8.2	0.7	7.8	5.1	0.5	1.6	2.7	0.5	0.2	0.0	4.1	18.3	5.5	6.1	7.9
Bacteria; *Cyanobacteria*	7.2	0.1	2.8	7.6	0.1	4.8	0.0	0.3	0.0	5.5	2.6	1.9	2.4	9.1	2.4
Bacteria; *Planctomycetes*	6.8	0.6	5.6	4.2	0.4	0.5	0.5	0.4	0.1	1.6	0.7	2.4	0.6	1.8	1.3
Bacteria; *Actinobacteria*	3.5	0.6	3.7	3.2	1.1	0.9	0.2	0.7	0.1	4.2	1.5	2.5	0.9	0.6	2.2
Bacteria; *Firmicutes*	1.6	0.8	0.6	1.9	4.0	0.9	0.1	3.4	0.0	0.0	0.3	0.4	0.1	0.3	0.4
Bacteria; *Deinococcus-Thermus*	2.2	0.0	0.9	1.6	0.0	0.1	0.0	0.0	0.0	0.0	0.0	0.0	0.0	0.0	0.0
Archaea; *Euryarchaeota*	0.0	0.0	0.0	0.0	0.0	0.0	0.0	0.0	0.0	2.1	0.4	0.0	0.1	0.0	0.1
Unassigned and other phyla	6.6	34.0	8.1	4.1	39.2	1.5	59.5	49.6	0.1	0.2	1.2	0.7	0.4	0.8	1.1

42

Supplementary Table 5. Abundance (%) of major families in each samples. Families with >3.0% relative abundance in at least one sample are shown. Minor or unassigned families are shown as "Unassigned and other families".

	CF (*A. amurensis*)																		
	2014 N-MCF-1	2015 N-MCF-E	2015 N-MCF-D	2016N-MCF-M	2016 N-MCF-B	2015 N-MCF-C	2014U-MCF-Bulk	2016 N-MCF-F	2014 N-MCF-2	2016 M-MCF-A	2016 M-MCF-C	2016 N-MCF-C	2016 N-MCF-I	2016 N-MCF-N	2016 N-MCF-H	2016 N-MCF-K	2016 N-MCF-J	2016 N-MCF-L	2016 N-MCF-G
Bacteria; *Proteobacteria*; *Gammaproteobacteria*; *Thiotrichales*; *Thiotrichales* Incertae Sedis	0.1	1.1	1.6	0.3	0.1	0.1	0.5	0.5	1.0	0.0	0.0	95.6	75.0	98.4	69.7	90.2	2.6	40.1	95.4
Bacteria; *Proteobacteria*; *Epsilonproteobacteria*; *Campylobacterales*; *Helicobacteraceae*	11.0	90.2	54.5	26.9	97.3	89.4	0.6	0.1	39.6	7.1	9.1	0.1	21.9	0.1	3.1	8.6	15.6	16.7	0.1
Bacteria; *Tenericutes*; *Mollicutes*; *Mycoplasmatales*; *Mycoplasmataceae*	0.0	0.1	7.1	0.6	0.0	0.1	57.4	0.0	0.1	0.0	0.0	0.0	0.0	0.0	0.0	0.0	0.4	0.0	0.0
Bacteria; *Bacteroidetes*; *Flavobacteriia*; *Flavobacteriales*; *Flavobacteriaceae*	1.1	0.6	0.7	2.8	0.9	1.1	7.1	18.7	1.9	11.1	30.3	0.2	0.5	0.3	1.0	0.2	3.4	0.4	0.5
Bacteria; *Proteobacteria*; *Alphaproteobacteria*; *Rhodobacterales*; *Rhodobacteraceae*	0.1	0.4	0.3	0.2	0.1	0.3	5.7	13.5	2.3	10.1	14.9	0.2	0.2	0.0	0.1	0.1	1.4	0.3	0.2
Bacteria; *Proteobacteria*; *Epsilonproteobacteria*; *Campylobacterales*; *Campylobacteraceae*	0.0	0.0	0.1	0.0	0.0	0.0	0.3	0.1	0.2	2.4	0.0	0.0	0.0	0.0	0.0	0.0	0.3	0.0	0.0
Bacteria; *Proteobacteria*; *Betaproteobacteria*; *Nitrosomonadales*; *Nitrosomonadaceae*	0.0	0.1	0.1	0.0	0.0	0.1	0.0	0.0	0.1	0.0	0.0	0.0	0.0	0.0	0.0	0.0	0.0	0.0	0.0
Bacteria; *Proteobacteria*; *Gammaproteobacteria*; *Alteromonadales*; *Pseudoalteromonadaceae*	1.2	0.1	0.0	0.4	0.0	0.2	0.3	2.5	0.7	0.1	1.6	0.0	0.2	0.3	0.3	0.0	3.4	0.0	0.3
Bacteria; *Verrucomicrobia*; *Verrucomicrobiae*; *Verrucomicrobiales*; *Verrucomicrobiaceae*	2.7	0.1	0.1	0.1	0.0	0.1	0.2	2.4	0.6	0.9	0.6	0.0	0.0	0.0	0.0	0.0	0.0	0.0	0.0
Bacteria; *Proteobacteria*; *Gammaproteobacteria*; *Oceanospirillales*; *Alcanivoracaceae*	0.0	0.0	0.0	0.0	0.0	0.0	0.1	5.6	0.0	0.0	0.0	0.0	0.9	0.0	0.7	0.0	14.0	0.4	0.6
Bacteria; *Proteobacteria*; *Gammaproteobacteria*; *Vibrionales*; *Vibrionaceae*	0.3	0.1	0.5	0.1	0.0	0.3	1.6	0.4	6.0	2.3	1.2	0.0	0.0	0.0	0.0	0.0	0.2	0.0	0.0
Bacteria; *Bacteroidetes*; *Flavobacteriia*; *Flavobacteriales*; *Cryomorphaceae*	0.0	0.1	0.2	0.1	0.0	0.1	5.9	0.9	0.1	1.2	0.9	0.0	0.1	0.1	0.1	0.1	0.6	0.0	0.0
Bacteria; *Proteobacteria*; *Alphaproteobacteria*; *Sphingomonadales*; *Erythrobacteraceae*	0.0	0.0	0.0	0.0	0.0	0.0	0.8	8.5	0.0	0.3	1.3	0.0	0.0	0.0	0.1	0.0	0.9	0.0	0.1
Bacteria; *Proteobacteria*; *Betaproteobacteria*; *Burkholderiales*; *Comamonadaceae*	0.0	0.1	0.1	0.2	0.0	0.1	0.0	0.0	0.1	0.0	0.0	0.0	0.0	0.0	0.0	0.1	0.0	0.1	0.0
Bacteria; *Proteobacteria*; *Gammaproteobacteria*; *Cellvibrionales*; *Halieaceae*	0.1	0.0	0.1	0.1	0.4	0.0	0.5	1.5	0.2	1.7	1.6	0.2	0.0	0.0	0.1	0.0	0.3	0.2	0.1
Bacteria; *Proteobacteria*; *Gammaproteobacteria*; *Alteromonadales*; *Idiomarinaceae*	0.0	0.0	0.0	0.0	0.0	0.0	1.4	6.2	0.0	0.0	0.0	0.0	0.2	0.0	1.0	0.0	4.8	0.1	0.8
Bacteria; *Cyanobacteria*; *Cyanobacteria*; Subsection III; Family I	0.0	0.0	0.0	0.0	0.0	0.0	0.0	1.1	0.1	0.0	0.0	0.0	0.0	0.0	0.0	0.0	0.0	0.0	0.0
Bacteria; *Cyanobacteria*; *Chloroplast*; Other; Other	0.0	0.0	0.0	0.0	0.0	0.0	0.0	0.2	0.1	0.3	1.6	0.0	0.0	0.0	0.0	0.0	0.1	0.0	0.0
Bacteria; *Bacteroidetes*; *Sphingobacteriia*; *Sphingobacteriales*; *Saprospiraceae*	0.0	0.1	0.1	0.1	0.0	0.0	0.1	2.6	0.1	1.6	1.6	0.0	0.1	0.0	0.1	0.1	0.9	0.0	0.1
Bacteria; *Proteobacteria*; *Alphaproteobacteria*; *Sphingomonadales*; *Sphingomonadaceae*	1.5	0.1	0.0	0.1	0.1	0.1	0.4	4.8	0.1	1.3	1.3	0.1	0.0	0.0	0.0	0.0	0.2	0.1	0.0
Bacteria; *Cyanobacteria*; *Chloroplast*; Ambiguous taxa;	0.0	0.0	0.0	0.0	0.0	0.0	0.0	0.0	0.0	0.1	0.0	0.0	0.0	0.0	0.0	0.0	0.0	0.0	0.0
Bacteria; *Bacteroidetes*; *Cytophagia*; *Cytophagales*; *Cytophagaceae*	0.0	0.0	0.0	0.1	0.0	0.0	0.0	0.6	0.2	0.5	0.0	0.0	0.0	0.0	0.0	0.0	0.0	0.0	0.0
Bacteria; *Proteobacteria*; *Gammaproteobacteria*; *Alteromonadales*; *Colwelliaceae*	0.0	0.1	0.1	0.0	0.0	0.0	0.1	2.3	0.0	0.4	0.0	0.0	0.0	0.0	0.0	0.0	0.0	0.0	0.0
Bacteria; *Proteobacteria*; *Gammaproteobacteria*; *Aeromonadales*; *Aeromonadaceae*	0.0	0.0	0.0	0.0	0.0	0.0	0.3	1.2	0.1	0.0	0.0	0.0	0.0	0.0	0.0	0.0	0.0	0.0	0.0
Bacteria; Planctomycetes; Planctomycetacia; Planctomycetales; Planctomycetaceae	3.0	0.3	0.3	0.0	0.0	0.3	0.5	1.2	0.3	0.5	0.0	0.0	0.0	0.0	0.0	0.0	0.2	0.0	0.0
Bacteria; *Proteobacteria*; *Gammaproteobacteria*; *Pseudomonadales*; *Pseudomonadaceae*	0.0	0.0	0.0	0.0	0.0	0.0	0.0	0.0	0.0	0.0	0.5	0.0	0.0	0.0	0.0	0.0	0.0	0.0	0.0
Bacteria; *Firmicutes*; *Bacilli*; *Bacillales*; *Staphylococcaceae*	0.4	0.0	0.0	0.1	0.0	0.0	0.0	0.2	3.7	0.0	0.0	0.3	0.0	0.0	0.0	0.0	0.0	0.2	0.1
Bacteria; Proteobacteria; Gammaproteobacteria; *Oceanospirillales*; SAR86 clade	0.0	0.0	0.0	0.0	0.1	0.0	0.1	0.3	0.0	0.2	1.0	0.1	0.0	0.0	0.1	0.0	0.5	0.0	0.0
Bacteria; *Actinobacteria*; *Acidimicrobiia*; *Acidimicrobiales*; OM1 clade	0.4	0.0	0.0	0.0	0.0	0.0	0.0	0.0	0.0	0.0	0.0	0.0	0.0	0.0	0.0	0.0	0.0	0.0	0.0
Bacteria; *Proteobacteria*; *Gammaproteobacteria*; *Thiotrichales*; *Piscirickettsiaceae*	0.0	0.0	0.1	0.0	0.0	0.0	0.0	0.0	0.0	0.0	0.0	0.0	0.0	0.0	0.0	0.0	0.0	0.0	0.0
Bacteria; *Proteobacteria*; *Deltaproteobacteria*; *Bdellovibrionales*; *Bdellovibrionaceae*	0.0	0.0	0.0	0.0	0.0	0.0	0.1	0.1	0.0	0.3	0.0	0.0	0.0	0.0	0.0	0.0	0.0	0.0	0.0
Bacteria; *Bacteroidetes*; *Sphingobacteriia*; *Sphingobacteriales*; uncultured	0.0	0.0	0.0	0.1	0.0	0.0	0.0	0.1	0.0	0.0	0.0	0.0	0.0	0.0	0.0	0.0	0.0	0.0	0.0
Unassigned and other families	77.8	6.1	33.9	67.6	0.9	7.5	15.9	24.7	42.0	57.7	32.6	3.3	0.9	0.7	23.5	0.6	50.1	41.4	1.5

	CF (*P. pectinifera*)									Seawater					Starfish body surface
	2015N-ICF-Bulk	2016 N-ICF-A	2015 N-ICF-B	2015 N-ICF-A	2016 N-ICF-B	2016 U-ICF-B	2016 U-ICF-A	2016 N-ICF-D	2016 U-ICF-2	2016N-SW	2015 N-SW-3	2015 N-SW-1	2015H-SW	2015 N-SW-2	2015N-MS-A
Bacteria; *Proteobacteria*; *Gammaproteobacteria*; *Thiotrichales*; *Thiotrichales* Incertae Sedis	0.0	0.0	0.0	0.1	0.1	0.6	0.0	0.8	0.0	0.0	0.0	0.0	0.0	0.0	0.0
Bacteria; *Proteobacteria*; *Epsilonproteobacteria*; *Campylobacterales*; *Helicobacteraceae*	0.8	6.9	1.3	6.2	4.1	1.6	0.5	2.7	0.0	0.7	3.8	8.2	0.3	2.4	4.3
Bacteria; *Tenericutes*; *Mollicutes*; *Mycoplasmatales*; *Mycoplasmataceae*	0.0	0.0	0.0	0.0	0.0	0.0	0.0	0.0	92.3	0.0	0.0	0.0	0.0	0.0	0.0
Bacteria; *Bacteroidetes*; *Flavobacteriia*; *Flavobacteriales*; *Flavobacteriaceae*	10.4	7.4	12.4	7.8	7.8	19.3	5.1	7.5	1.2	10.5	10.3	3.2	19.5	9.1	12.6
Bacteria; *Proteobacteria*; *Alphaproteobacteria*; *Rhodobacterales*; *Rhodobacteraceae*	14.5	5.6	16.4	15.0	2.9	12.2	13.9	2.7	2.0	2.9	13.5	11.6	29.0	4.5	10.3
Bacteria; *Proteobacteria*; *Epsilonproteobacteria*; *Campylobacterales*; *Campylobacteraceae*	0.2	3.3	0.3	0.9	1.8	22.4	0.5	4.5	0.3	1.9	5.7	1.0	0.4	0.6	1.6
Bacteria; *Proteobacteria*; *Betaproteobacteria*; *Nitrosomonadales*; *Nitrosomonadaceae*	0.5	17.0	0.5	2.2	20.6	2.1	1.1	9.0	0.3	0.0	0.0	0.0	0.0	0.0	0.0
Bacteria; *Proteobacteria*; *Gammaproteobacteria*; *Alteromonadales*; *Pseudoalteromonadaceae*	0.1	0.6	0.3	0.5	0.4	1.6	1.2	0.1	0.1	20.5	0.4	0.1	4.9	4.3	0.3
Bacteria; *Verrucomicrobia*; *Verrucomicrobiae*; *Verrucomicrobiales*; *Verrucomicrobiaceae*	6.4	0.3	4.7	3.0	0.1	0.6	0.9	0.2	0.1	0.0	2.9	17.1	4.0	5.3	6.9
Bacteria; *Proteobacteria*; *Gammaproteobacteria*; *Oceanospirillales*; *Alcanivoracaceae*	0.0	1.5	0.1	0.0	0.2	0.0	0.0	0.6	0.0	0.1	0.1	0.0	0.0	0.0	0.0
Bacteria; *Proteobacteria*; *Gammaproteobacteria*; *Vibrionales*; *Vibrionaceae*	2.3	0.8	1.3	1.7	0.2	0.8	0.3	0.1	0.2	12.5	2.7	1.1	1.3	4.3	1.3
Bacteria; *Bacteroidetes*; *Flavobacteriia*; *Flavobacteriales*; *Cryomorphaceae*	1.0	0.7	1.4	1.5	0.9	1.1	0.0	0.8	0.1	2.7	6.6	8.5	8.9	6.7	10.9
Bacteria; *Proteobacteria*; *Alphaproteobacteria*; *Sphingomonadales*; *Erythrobacteraceae*	3.0	0.5	0.8	0.4	0.4	1.5	1.4	0.0	0.0	0.0	0.0	0.1	0.0	0.1	0.0
Bacteria; *Proteobacteria*; *Betaproteobacteria*; *Burkholderiales*; *Comamonadaceae*	0.1	0.0	0.0	0.3	0.0	0.0	0.0	0.3	0.0	0.4	0.0	0.0	0.2	7.1	0.1
Bacteria; *Proteobacteria*; *Gammaproteobacteria*; *Cellvibrionales*; *Halieaceae*	1.0	2.1	2.7	2.2	1.4	1.8	1.0	2.4	0.3	4.3	3.8	6.3	2.1	2.5	4.9
Bacteria; *Proteobacteria*; *Gammaproteobacteria*; *Alteromonadales*; *Idiomarinaceae*	0.0	0.7	0.0	0.0	0.1	0.1	0.0	2.7	0.0	0.0	0.0	0.0	0.3	0.0	0.0
Bacteria; *Cyanobacteria*; *Cyanobacteria*; Subsection III; Family I	6.1	0.0	0.9	1.8	0.0	0.0	0.0	0.0	0.0	2.4	0.0	0.0	0.0	0.0	0.0
Bacteria; *Cyanobacteria*; *Chloroplast*; Other; Other	0.1	0.0	0.2	1.9	0.0	0.1	0.0	0.1	0.0	1.3	1.3	0.2	1.9	6.0	1.1
Bacteria; *Bacteroidetes*; *Sphingobacteriia*; *Sphingobacteriales*; *Saprospiraceae*	1.8	0.1	1.4	0.9	0.8	0.8	0.4	0.4	0.1	5.6	0.6	1.3	2.5	3.0	1.2
Bacteria; *Proteobacteria*; *Alphaproteobacteria*; *Sphingomonadales*; *Sphingomonadaceae*	1.5	0.3	1.1	0.6	0.6	0.6	1.3	1.3	0.1	0.0	0.1	0.0	0.0	0.3	0.0
Bacteria; *Cyanobacteria*; *Chloroplast*; Ambiguous taxa;	0.2	0.0	0.2	1.5	0.0	4.6	0.0	0.0	0.0	0.0	0.1	0.0	0.1	0.4	0.1
Bacteria; *Bacteroidetes*; *Cytophagia*; *Cytophagales*; *Cytophagaceae*	4.5	0.3	4.0	4.0	0.1	0.1	0.1	0.0	0.0	0.0	0.0	0.0	0.0	1.4	0.0
Bacteria; *Proteobacteria*; *Gammaproteobacteria*; *Alteromonadales*; *Colwelliaceae*	0.0	4.4	0.1	0.2	1.6	1.2	0.1	0.0	0.0	2.7	0.6	0.2	0.4	0.2	0.2
Bacteria; *Proteobacteria*; *Gammaproteobacteria*; *Aeromonadales*; *Aeromonadaceae*	0.0	0.0	0.0	0.0	0.0	0.2	0.0	0.0	0.0	4.3	0.0	0.0	0.1	0.5	0.0
Bacteria; Planctomycetes; Planctomycetacia; Planctomycetales; Planctomycetaceae	4.0	0.5	4.0	3.0	0.4	0.4	0.5	0.3	0.0	0.1	0.4	1.4	0.2	0.7	0.5
Bacteria; *Proteobacteria*; *Gammaproteobacteria*; *Pseudomonadales*; *Pseudomonadaceae*	0.0	0.0	0.0	0.1	0.0	0.0	0.0	0.0	0.0	3.8	0.0	0.0	0.0	0.9	0.0
Bacteria; *Firmicutes*; *Bacilli*; *Bacillales*; *Staphylococcaceae*	0.3	0.6	0.0	0.2	3.7	0.3	0.0	2.8	0.0	0.0	0.0	0.0	0.0	0.0	0.0
Bacteria; Proteobacteria; Gammaproteobacteria; *Oceanospirillales*; SAR86 clade	0.1	0.0	0.0	0.3	0.1	0.1	0.0	0.1	0.0	0.0	3.1	2.2	2.0	0.2	3.3
Bacteria; *Actinobacteria*; *Acidimicrobiia*; *Acidimicrobiales*; OM1 clade	0.0	0.0	0.1	0.1	0.0	0.0	0.0	0.0	0.0	3.2	0.6	0.9	0.6	0.1	1.1
Bacteria; *Proteobacteria*; *Gammaproteobacteria*; *Thiotrichales*; *Piscirickettsiaceae*	0.2	0.1	0.3	0.8	0.0	0.2	0.0	0.0	0.0	0.3	3.2	2.7	0.2	0.8	1.7
Bacteria; *Proteobacteria*; *Deltaproteobacteria*; *Bdellovibrionales*; *Bdellovibrionaceae*	0.2	0.1	0.4	0.5	0.1	0.4	0.2	0.2	0.0	1.7	1.1	2.4	0.4	3.1	2.1
Bacteria; *Bacteroidetes*; *Sphingobacteriia*; *Sphingobacteriales*; uncultured	0.2	0.0	0.1	0.4	0.1	0.1	0.0	0.0	0.0	0.4	0.6	1.4	0.3	3.1	1.4
Unassigned and other families	40.3	46.0	45.2	41.7	51.5	25.2	71.4	60.3	2.7	17.7	38.6	29.9	20.3	32.5	33.8

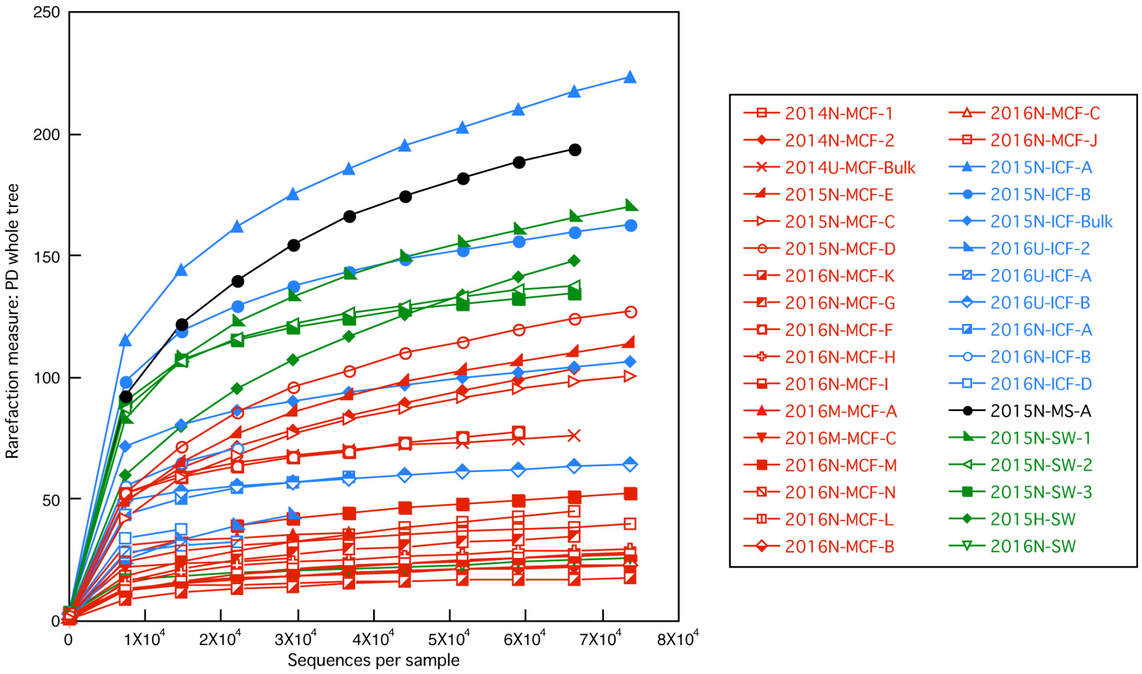

Supplementary Figure 1. Rarefaction curves for OTUs (97% sequence similarity cut-off). *A. amurensis* CF samples, *P. pectinifera* CF samples, seawater, and starfish body surface samples are shown in red, blue, green, and black, respectively.

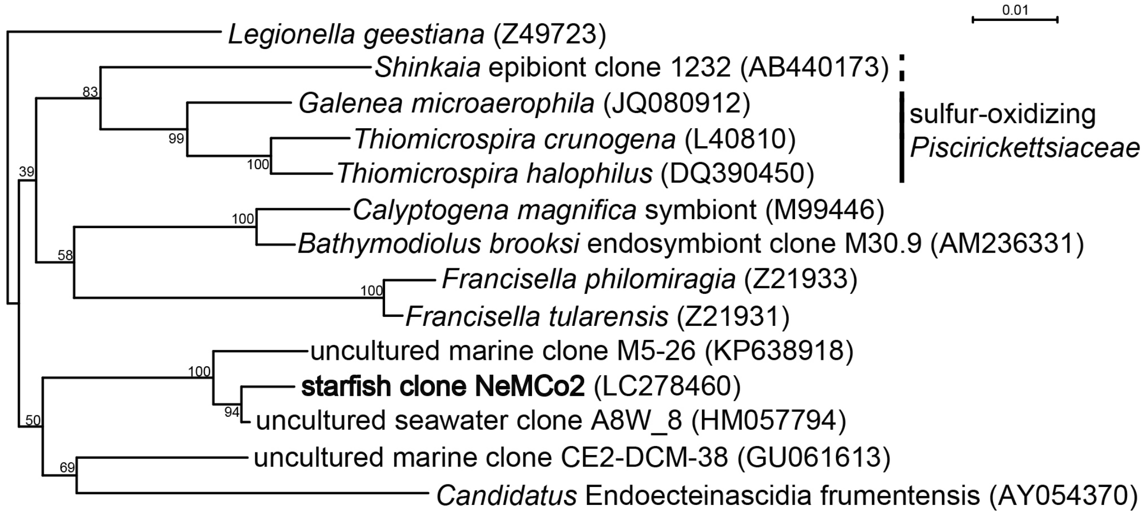

Supplementary Figure 2. Phylogenetic tree of representative members of the order *Thiotrichales* inferred from 16S rRNA gene sequences by the neighbour joining method using 962 homologous sequence positions. The accession numbers are shown in parentheses. Bootstrap values (expressed as percentages of 1,000 replications) are shown at branching points. The sequence found in the CF of *A. amurensis* is shown in bold. The scale bar represents 0.01 substitutions per nucleotide position.
